# Acceleration-Based Kalman Tracking for Super-Resolution Ultrasound Imaging *in vivo*

**DOI:** 10.1109/TUFFC.2023.3326863

**Published:** 2023-10-23

**Authors:** Biao Huang, Jipeng Yan, Megan Morris, Victoria Sinnett, Navita Somaiah, Meng-Xing Tang

**Affiliations:** Ultrasound Lab for Imaging and Sensing, Department of Bioengineering, Imperial College London, London, UK, SW7 2AZ; Ultrasound Lab for Imaging and Sensing, Department of Bioengineering, Imperial College London, London, UK, SW7 2AZ; Ultrasound Lab for Imaging and Sensing, Department of Bioengineering, Imperial College London, London, UK, SW7 2AZ; Royal Marsden NHS Foundation Trust, London, UK, SW3 6JJ; Royal Marsden NHS Foundation Trust and The Institute of Cancer Research, London, UK, SM2 5NG; Ultrasound Lab for Imaging and Sensing, Department of Bioengineering, Imperial College London, London, UK, SW7 2AZ

**Keywords:** Kalman filter, medical imaging, microbubbles, microvasculature, motion model, ultrasound localisation microscopy

## Abstract

Super-resolution ultrasound can image microvascular structure and flow at sub-wave-diffraction resolution based on localising and tracking microbubbles. Currently, tracking microbubbles accurately under limited imaging frame rates and high microbubble concentrations remains a challenge, especially under the effect of cardiac pulsatility and in highly curved vessels. In this study, an acceleration-incorporated microbubble motion model is introduced into a Kalman tracking framework. The tracking performance was evaluated using simulated microvasculature with different microbubble motion parameters, concentrations and acquisition frame rates, and *in vivo* human breast tumour ultrasound datasets. The simulation results show that the acceleration-based method outperformed the non-acceleration-based method at different levels of acceleration and acquisition frame rates and achieved significant improvement in true positive rate (up to 11.3%), false negative rate (up to 13.2%). The proposed method can also reduce errors in vasculature reconstruction via the acceleration-based nonlinear interpolation, compared with linear interpolation (up to 16.7 *μ*m). The tracking results from temporally downsampled low frame rate *in vivo* datasets from human breast tumours show that the proposed method has better microbubble tracking performance than the baseline method, if using results from the initial high frame data as reference. Finally, the acceleration estimated from tracking results also provides a spatial speed gradient map that may contain extra valuable diagnostic information.

## Graphical abstract

**Figure:**
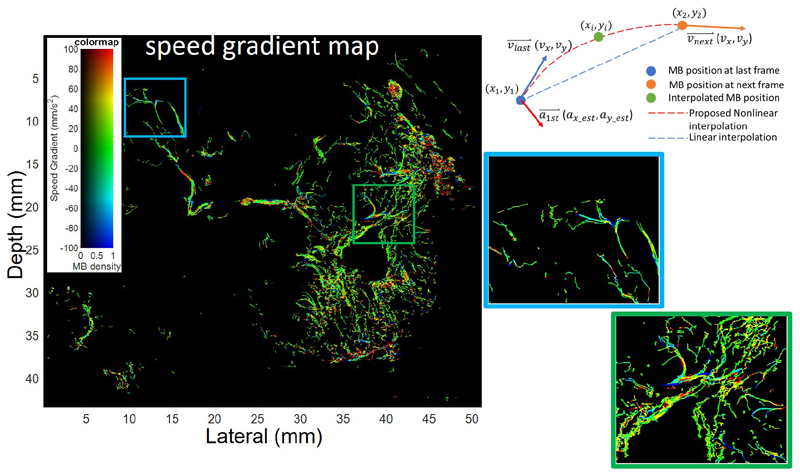

## I Introduction

SUPER-resolution ultrasound (SRUS), also known as ultrasound localisation microscopy (ULM), based on localising and tracking sparse microbubbles (MBs), is capable of mapping microvasculature beyond the wave diffraction limit *in vitro*[1], [2] and *in vivo*[3]–[5]. Subsequently, the technique has been applied in a range of in vivo models, such as rat tumor angiogenesis [6], mouse acute kidney injury [7], rat kidney [8]–[10], rabbit kidney perfusion study [11], renal tumor in chicken embryo [12] and sheep ovary vasculature [13] and in human, including the breast [14], limbs [15], the liver [16], lymph nodes [17], and the brain [18].

Flows in microvasculature can be measured by SRUS via MB tracking, the performance of which is affected by acquisition frame rates and MB concentrations [19], [20]. As high frame rate acquisitions are not generally available in commercial US systems, a low MB concentration is often required to maintain tracking accuracy, leading to a long acquisition time to reconstruct vasculature [21]. More tissue motion might happen during a longer acquisition, which makes motion correction more challenging [15]. It is valuable to develop algorithms to track MBs at high concentrations and low frame rates. Various MB motion models have been used in SRUS to deal with the aforementioned problems. These algorithms are associated with detected MB positions based on a probabilistic optimisation. A Markov Chain Monte Carlo data association algorithm (MCMCDA) [14], [22] and multiply hypothesis tracking (MHT) procedure [23] was proposed to handle MB tracking at high MB concentration. Yet, one limitation of both methods is the relatively high computational cost. A multi-feature-based tracking algorithm was proposed in [20], whereas the linear motion model was combined with MB image features. Similarly, a hierarchical algorithm with Kalman filtering has also been developed to track MBs at different speed ranges [9]. However, all the aforementioned methods were using a linear motion model where MB movement between consecutive frames was assumed in constant velocity. However, vessels are with curvature and pulsating flow. A motion model with constant curvature radius and speed was proposed by [24], and an unscented Kalman filter was implemented to handle the model’s nonlinearity. Kalman filtering was also used in [19] to smooth the MB trajectory after MB pairing.

Movement of MBs are sampled by SRUS at the frame rate of acquisition, which can consequentially generate a discontinuity in estimated speed and/or direction of MB movement between frames in case of significant changes in flow velocity. In general, the problem of discontinuity is exacerbated by lower frame rates and faster flow speeds. In general, lower acquisition frame rates and faster flow speed exacerbate the problem of discontinuity, resulting in larger gaps between adjacent MB locations. The reconstruction of vasculature from the accumulation of MB locations in such situation is lack of saturation, *i.e*., portion of the vascular space filled by MBs, compared to the accumulation of continuous MB trajectories. Therefore, interpolating tracked MB locations between frames has been used to fill the gaps and enhance the visualisation quality by linking paired MBs with straight lines [20] or further adaptively changing distance between interpolated points [19]. However, the assumption that MBs were moving in straight lines between frames is not true for curved vessels. A nonlinear interpolation for MB trajectory reconstruction is worth exploring to provide a more accurate reconstruction of the microvasculature.

MB tracking allows dynamic flow parameters to be mapped at super-resolution, such as flow speed and direction [3], which adds significant value to potential clinical applications of SRUS. Opacic [14] and Zhu [25] have shown that regularity of microvascular flow directions can be a potential marker for cancer in human. A previous clinical study of breast cancer found that the acceleration time index is a useful parameter for differentiating benign breast tumours from malignant tumours by using Doppler ultrasonography [26].

In this study, based on the assumption that MBs may travel in non-straight vessels and may have non-zero acceleration, we aim to improve the MB tracking algorithm by incorporating an acceleration term in the current Kalman filtering framework, to account for changes of flow speed and direction between frames. Curved trajectories of MBs were reconstructed via Kalman state vectors. A spatial speed gradient map calculated from acceleration was presented.

## II Methods

This section firstly describes the acceleration incorporated Kalman tracking framework and a 3D graph-based method for initialising the velocity of new MBs. Next, the nonlinear MB trajectory interpolation method based on estimated acceleration was described. Finally, the proposed method was evaluated on both simulation and *in vivo* datasets.

### A Acceleration-based Kalman Tracking with 3-frame Initialisation

MBs can be effectively tracked via the graph-based assignment framework [19]. We have recently developed a framework which pairs MBs between two consecutive frames by minimising the total cost constructed by image features and a linear motion model [20]. The linear motion model was used to predict the movement of MBs, where each MB is assumed to move at a constant velocity between two adjacent frames. This assumption is not valid when MB moves in a curved vessel and flow acceleration is significant, especially when the acquisition frame rate is low. A more accurate motion model is required.

A nonlinear motion-based Kalman filtering has been applied to MB tracking, where the motion model is incorporated as part of the MB tracking cost. To be more specific, a probability (p) that indicates the likelihood of an MB pair between frames is defined as a cost, given below

(1) $$Cost_{track}=1/p=1/N(\mu ,\text{Σ})$$

(2) $$\mu =H_{k}\times S_{k|k-1}$$

(3) $$\text{Σ}=H_{k}\times P_{k|k-1}\times H_{k}^{T}+R_{k}$$

where *N* is defined by the Gaussian distribution, *H_k_* and *R_k_* are the observation model and the covariance of observation noise, respectively, *S*_*k*|*k*-1_ and *P*_*k*\*k*-1_ are the predicted state vector and predicted estimate covariance matrix, respectively, and are both predicted by the state transition matrix *F*. The state transition model is shown in Eq. (4) and (5).

(4) $$S_{k|k-1}=F\times S_{k-1|k-1}$$

(5) $$\begin{array}{ccccc} \left[\begin{array}{c} x(k)\\ v_{x}(k)\\ a_{x}(k)\\ y(k)\\ v_{y}(k)\\ a_{y}(k) \end{array}\right] & = & \left[\begin{array}{cccccc} 1 & \text{Δ}t & \frac{\text{Δ}t^{2}}{2} & 0 & 0 & 0\\ 0 & 1 & \text{Δ}t & 0 & 0 & 0\\ 0 & 0 & 1 & 0 & 0 & 0\\ 0 & 0 & 0 & 1 & \text{Δ}t & \frac{\text{Δ}t^{2}}{2}\\ 0 & 0 & 0 & 0 & 1 & \text{Δ}t\\ 0 & 0 & 0 & 0 & 0 & 1 \end{array}\right] & \times & \left[\begin{array}{c} x(k-1)\\ v_{x}(k-1)\\ a_{x}(k-1)\\ y(k-1)\\ v_{y}(k-1)\\ a_{y}(k-1) \end{array}\right] \end{array}$$

In this study, we modelled the MB movement between frames as an accelerated motion. Thus, the state *S* contains two-dimensional MB location (*x,y*), velocity (*v_x_,v_y_*) and acceleration (*a_x_, a_y_*). Δ*t* is the time interval between frames. *Q* is the covariance of processing noise, which is used to describe the uncertainty of the true motion from the motion model. $\sigma_{a}^{2}$ is the variance of noise when assuming constant acceleration between frames.

**Fig. 1:**
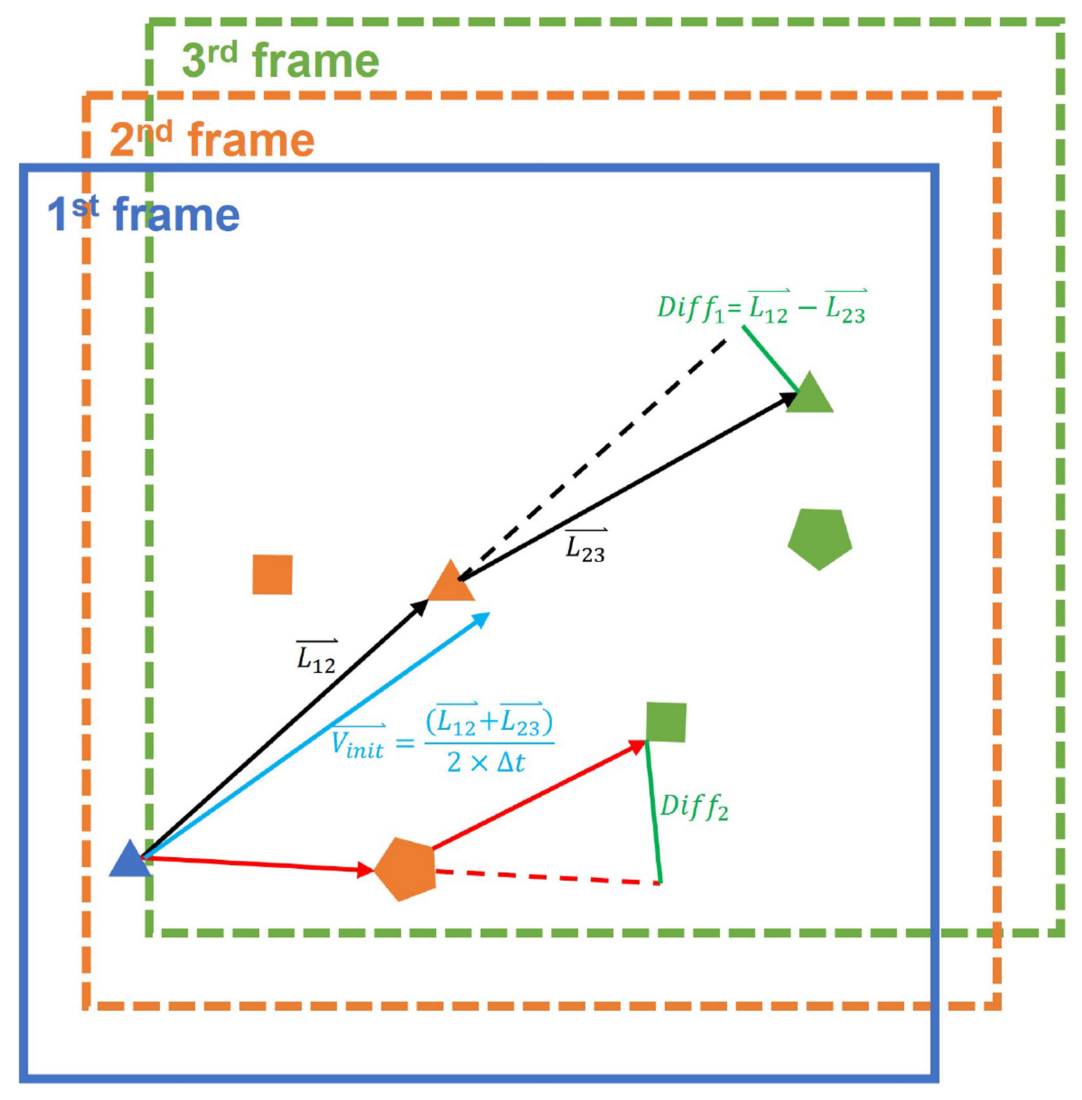
Illustration of 3-frame initialisation. The black line indicates the true MB pairing. The red line indicates the incorrect MB paring. Green lines indicate the vector difference calculated; the normalised difference is defined as the cost (Eq. (6)) for initialisation. The blue arrow indicates the velocity estimated from the initial MB pairs (Eq. (7)).

The state vector of a new MB, including its moving direction, is unknown and can be initiated by using multiple frame tracking. To enable motion model for the new MB, a 3-frame state initialisation method was proposed to give new MBs an initial guess of their states, rather than be set with zero velocity. An assumption for this initialisation is that MB moves smoothly between frames, which indicates the motion direction of MBs between frames will not change dramatically [27] (Fig. 1). Thus, the true MB pairs can be found by minimising a cost defined by the normalised vector difference among three frames. The cost and state initialisation are given below

(6) $$Cost_{init}=\frac{\left\|\vec{L_{23}}-\vec{L_{12}}\right\|}{\left\|\vec{L_{23}}\|+\|\vec{L_{12}}\right\|}$$

(7) $$\vec{v_{init}}=\frac{\vec{L_{12}}+\vec{L_{23}}}{2\times \text{Δ}t}$$

(8) $$\vec{a_{init}}=\frac{\vec{L_{23}}+\vec{L_{12}}}{\text{Δ}t^{2}}$$

where $\vec{L_{12}}$ and $\vec{L_{23}}$ are the position vectors between frames, $\vec{v_{init}}$ and $\vec{a_{init}}$ indicate the initialised velocity and acceleration vector. The cost was minimised via a 3D graph-based assignment algorithm adapted from a 2D one [28], where a topology constraint that each MB can only be paired with no more than one MB at the next frame was set. The motion parameters, including velocity and acceleration, were then initialised from the paired MBs. Only new MBs were paired in the 3D graph-based assignment to initiate their state vectors, and subsequently, all MBs in two consecutive frames were paired using the 2D graph-based method.

### B Acceleration-based Nonlinear Interpolation of MB Tracks

**Fig. 2:**
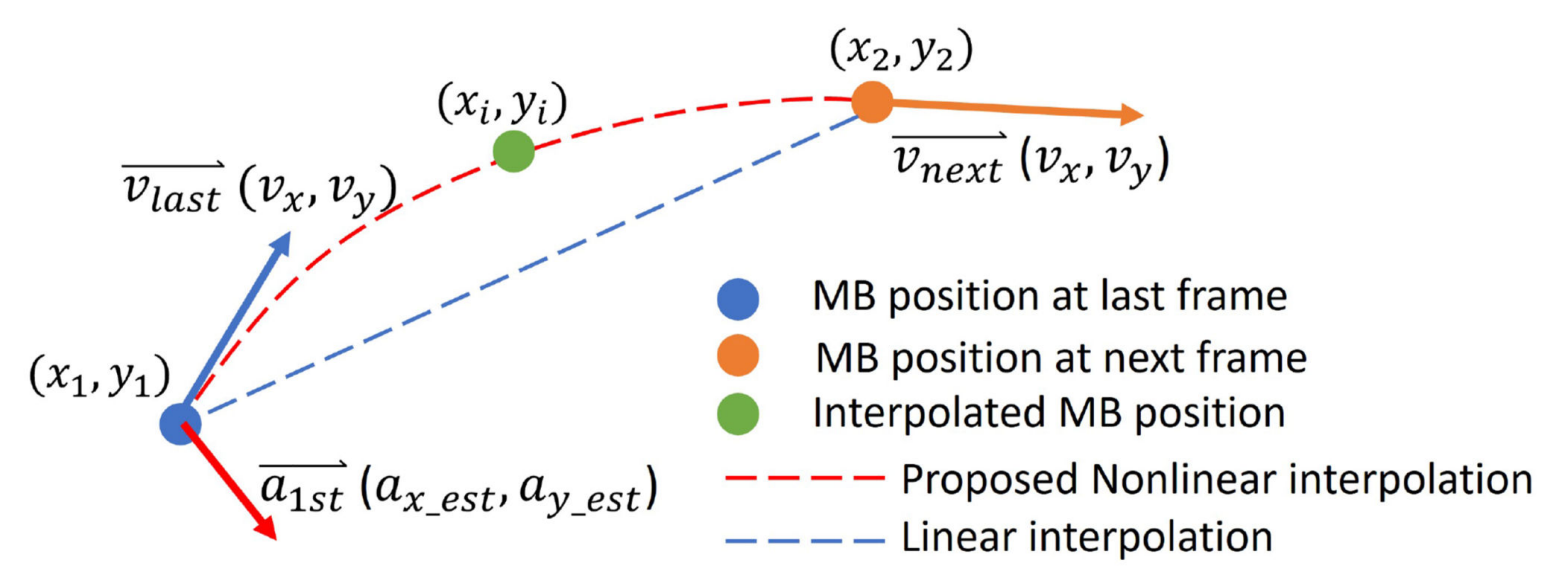
Examples of microvasculature simulation datasets for interpolation.

The microvasculature can be reconstructed by plotting tracked MBs. In this study, based on the estimated motion state from the Kalman-based tracking, we proposed a nonlinear interpolation method for MB trajectory reconstructions (Fig. 2).

The nonlinear interpolation used the MB’s acceleration estimated from Kalman states. The missing MB’s position between two frames was calculated following the motion model, given below

(9) $$\left\{ \begin{array}{l} x_{i}=x_{1}+v_{1x}\times dt+0.5\times a_{x_{-}est}\times dt^{2}\\ y_{i}=y_{1}+v_{1y}\times dt+0.5\times a_{y_{-}est}\times dt^{2} \end{array}\right.$$

where *x*_1_ and *y*_1_ indicate the starting coordinates of an MB, *v*_1*x*_ and *v*_1*y*_ are the estimated MB velocity from Kalman filtering, *a_x_est_* and *a_y_est_* are the estimated MB acceleration for interpolation, and *dt* is the time interval between the starting position and the position (*x_i_, y_i_*) that needs to be interpolated. The acceleration *a_x_est_* and *a_y_est_* are first calculated with Eq. (9) by replacing (*x_i_, y_i_*) with (*x*_2_, *y*_2_), where (*x*_2_, *y*_2_) is the MBs’ position at the next frame. The estimated acceleration (*a_x_est_, a_y_est_*) guarantees the continuous trajectory interpolation along all the paired MBs. The speed gradient for each MB can be calculated between the interpolated positions (*x_i_, y_i_*) and (*x*_*i*+1_, *y*_*i*+1_). The spatial speed gradient was then generated by averaging all the speed gradient at the same positions.

### C Evaluation via Simulations

The evaluation and comparison of the performance of acceleration-based MB tracking and trajectory interpolation methods are presented in this section. The algorithm and simulation dataset generation was implemented with MATLAB (R2022b, MathWorks, MA, USA).

#### 1 Evaluation of MB tracking

For the MB tracking performance comparison between models with and without acceleration components, we generated ten microvasculature simulation datasets. Each dataset had two main vessels each branching into another three downstream vessels. Three different acquisition frame rates (15 Hz, 25 Hz, and 35 Hz) and three different MB concentrations, estimated from the clinical dataset (2.54×10^7^ MBs/mL, 3.82×10^7^ MBs/mL, 6.36×10^7^ MBs/mL), were used in the simulation. To simulate the effect of pulsatile blood flow from cardiac cycles, we accelerated and decelerated the MBs periodically, around the flow speed of 3 mm/s according to a heart rate of 75 bpm, for 30 seconds. Four different flow accelerations were set: 0 mm/s^2^, 37.5 mm/s^2^, 75.0 mm/s^2^, 112.5 mm/s^2^ [29], and a total of 360 localisation datasets were used to evaluate the tracking performance.

Both the acceleration-based tracking method (proposed) and the non-acceleration-based method (baseline) were tested using a non-parametric two-related-sample test, *i.e*., the Wilcoxon Signed Rank Test, in SPSS (Version 28.0, IBM Corp, NY, USA) at a significance level of 0.017, value of which was obtained via dividing 0.05 by the number of null hypotheses, *i.e*., 3, after applying the Bonferroni correction. The null hypothesis for this test is that there is no significant difference in the median of tracking performance between the proposed nonlinear method and the baseline linear method. Both methods were evaluated under various acceleration, frame rate, and MB concentration settings. The metrics for evaluating the tracking performance evaluation for each simulation setting were based on [30] which has been used in a recent super-resolution ultrasound challenge (https://ultrasr.com) and includes true positive rate (TPR), false negative rate (FNR), defined as:

(10) $$TruePositiveRate:\frac{TP}{TP+FP}$$

(11) $$FalseNegativeRate:1-\frac{TP}{TP+FN}$$

where *TP* is the number of true positive MB pairs, *FP* is the number of false positive MB pairs, *FN* is the number of false negative MB pairs.

#### 2 Evaluation for MB trajectory interpolation

Six additional datasets, each containing a single vessel, were generated to test the performance of MB trajectory interpolation. In this simulation, only one MB was inside each vessel to ensure the tracking precision, moved at a constant speed of 30 mm/s. Images were captured at frame rates of 15 Hz, 25 Hz, and 35 Hz for 60 seconds to ensure enough MBs passed through the vessel. Two different interpolation methods, linear interpolation, and acceleration-based nonlinear interpolation, were used for comparison. The linear interpolation method plotted straight trajectories between linked MB positions from adjacent frames, while the acceleration-based method plotted curved trajectories additionally with MB velocities estimated by either a linear or nonlinear motion model. Corresponding accelerations were calculated using Eq. (9). The reconstruction error was calculated by a pointwise Euclidean distance between interpolated results and the ground truth.

### D In vivo experiment

Two ultrasound datasets of breast cancer patients were acquired at The Royal Marsden Hospital (London, UK), for a clinical trial (KORTUC Phase 2, ClinicalTrials.gov: NCT03946202) led by the The Institute of Cancer Research and The Royal Marsden NHS Foundation Trust. Ethics approval was granted by West of Scotland Research Ethics Committee (REC ref 20/WS/0019). The patients were informed by and signed on written contents. The ultrasound datasets were acquired using a Verasonics Vantage (Verasonics Inc., Kirkland, WA, USA) and a GE LE-12D probe (GE Healthcare, NY, USA) with a centre frequency of 5 MHz. 2.5 mL of SonoVue MBs (Bracco, Milan, Italy) were administered intravenously. 5 seconds of the dataset was used for the super-resolution processing. Images were acquired at a frame rate of 100 Hz using a mechanical index (MI) of 0.1. An amplitude modulation (AM) was used to generate contrast-enhanced ultrasound (CEUS) sequences.

Tissue motion in datasets were estimated from the B-mode sequences, reconstructed from the AM pulse, using a non-rigid registration algorithm. The CEUS sequences were corrected correspondingly [15], [31]. A moving-average across 11 frames around the frame of interest was subtracted from the sequence to remove remaining tissue signals. The movingaverage window size was chosen after considering the frame rate, the velocity of the slowest blood flow to be captured, and the rate of tissue motion. The datasets were smoothed spatially and temporally using a Gaussian filter, and logarithmically compressed. To further reduce noises, a noise only dataset was acquired by imaging air and subtracted from the dataset of interest. The MB signal was localised by peaks in the map obtained by normalised cross-correlation with an estimated point spread function.

The acceleration-based and non-acceleration-based tracking methods were compared. To evaluate the influence of frame rate only and maintain the same data size, downsampling was conducted by 1) extracting the dataset by a time interval of 4 frames into 4 subgroups, 2) tracking MBs in each subgroup, and 3) combining all the tracking results to generate the final super-resolution map. Taking the tracking results obtained at 100 Hz as references, the tracking performance was evaluated by the consistency between the 25 Hz and 100 Hz frame rate tracking results.

## III Results

### A Simulations

#### 1 MB tracking

**Fig. 3:**
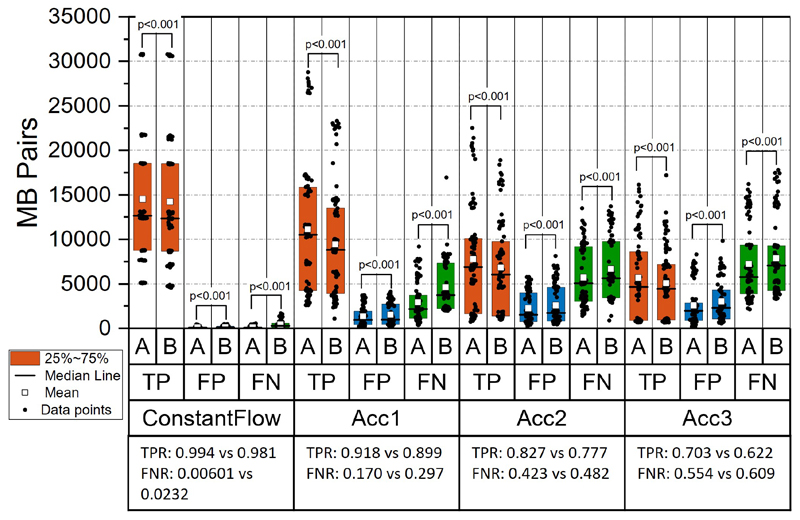
Paired-sample test between results from different accelerations. A: Proposed nonlinear model-based method. B: Baseline linear modelbased method. TP: true positive MB pairs. FP: false positive MB pairs. FN: false negative MB pairs. ConstantFlow: constant flow speed. Acci: simulation with an acceleration of 37.5 mm/s^2^. Acc2: 75.0 mm/s^2^. Acc3: 112.5 mm/s^2^. TPR: overall median true positive rate from proposed and baseline methods. FNR: overall median false negative rate.

**Fig. 4:**
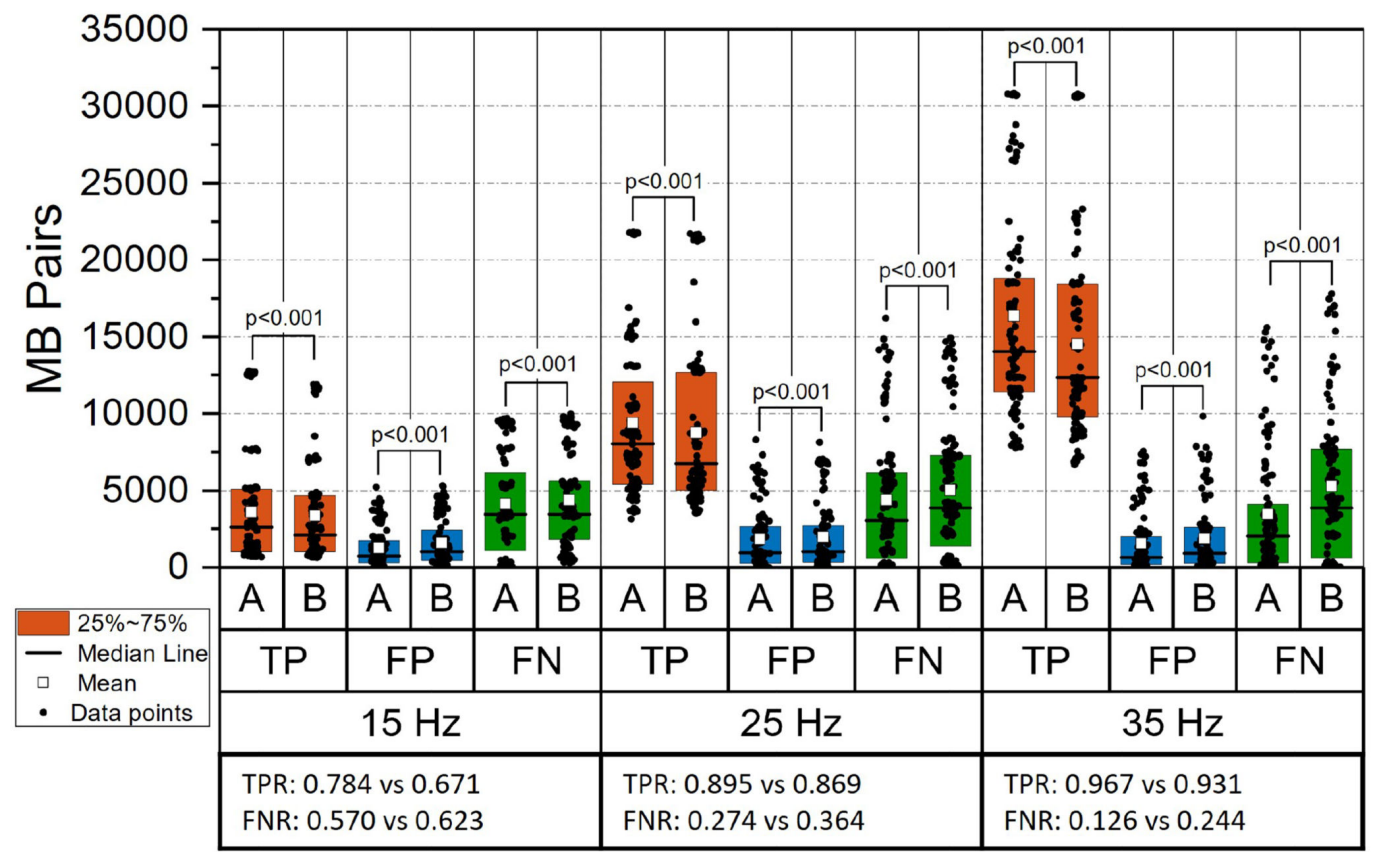
Paired-sample test between results from different acquisition frame rates. Captions are the same as in Fig 3.

**Fig. 5:**
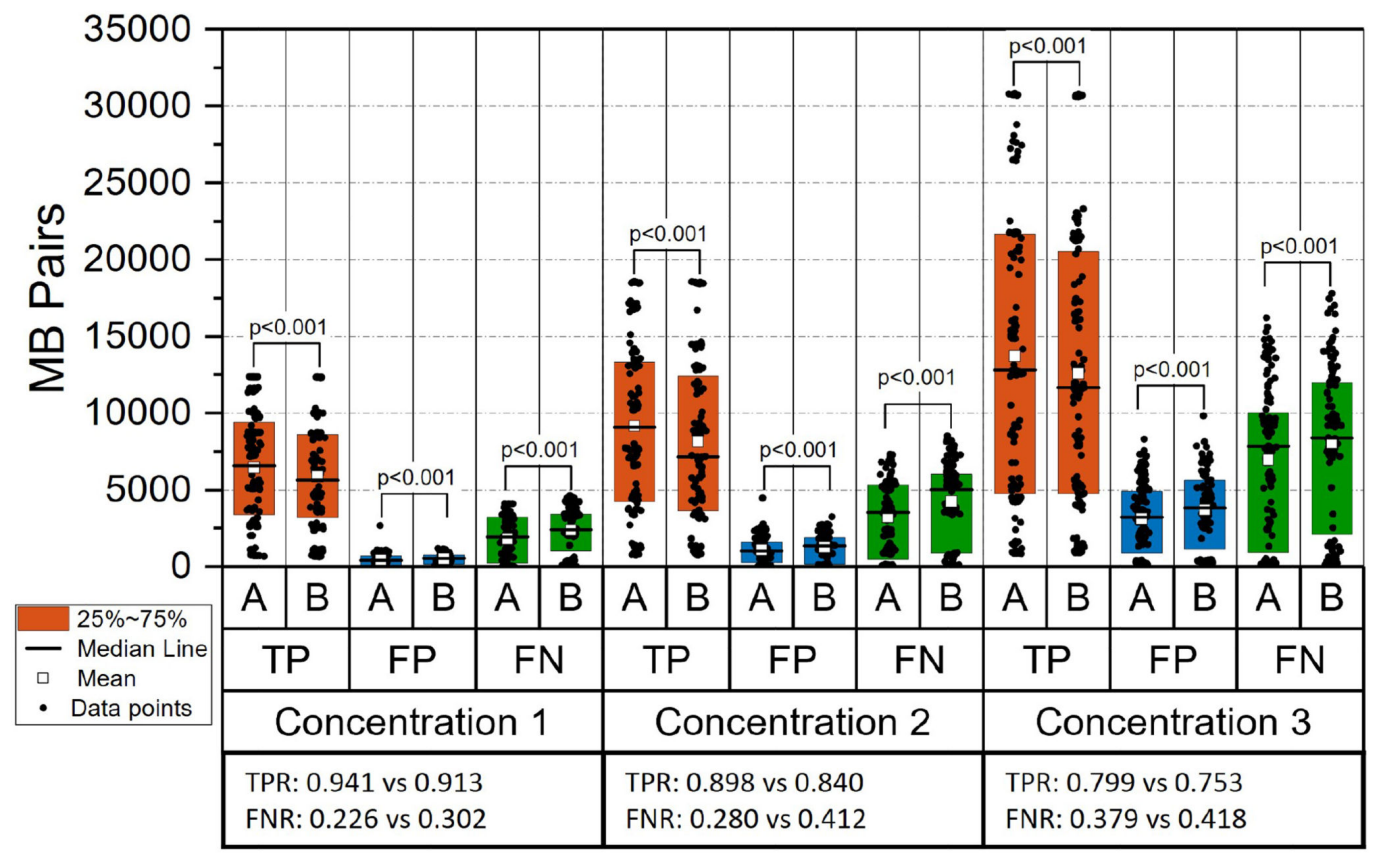
Paired-sample test between results from different MB concentrations. Captions are the same as in Fig 3. Concentration1: 2.54×10^7^ MBs/mL. Concentration2: 3.82×10^7^ MBs/mL. Concentration3: 6.36×10^7^ MBs/mL.

The evaluation results of MB tracking based on simulation data are shown in Fig. 3, Fig. 4 and Fig. 5. From the statistical analysis of MB tracking results, the proposed algorithm outperformed the baseline algorithm. We first evaluated the tracking performance of the proposed and baseline method on different acceleration settings (n=90 for each group). When there is no speed changing in the simulation, the results indicated there is still a small but significant improvement from the proposed method, as shown in Fig. 3. When there is an acceleration of MB moving speed, compared with the baseline method, the performance of both methods dropped, as the scenario became more challenging. The improvement of the proposed method is also significant under different frame rates and MB concentrations, as demonstrated in Fig. 4 and Fig. 5, indicated by higher number of TP pairs, lower number of FP and FN pairs with proposed method.

**Fig. 6:**
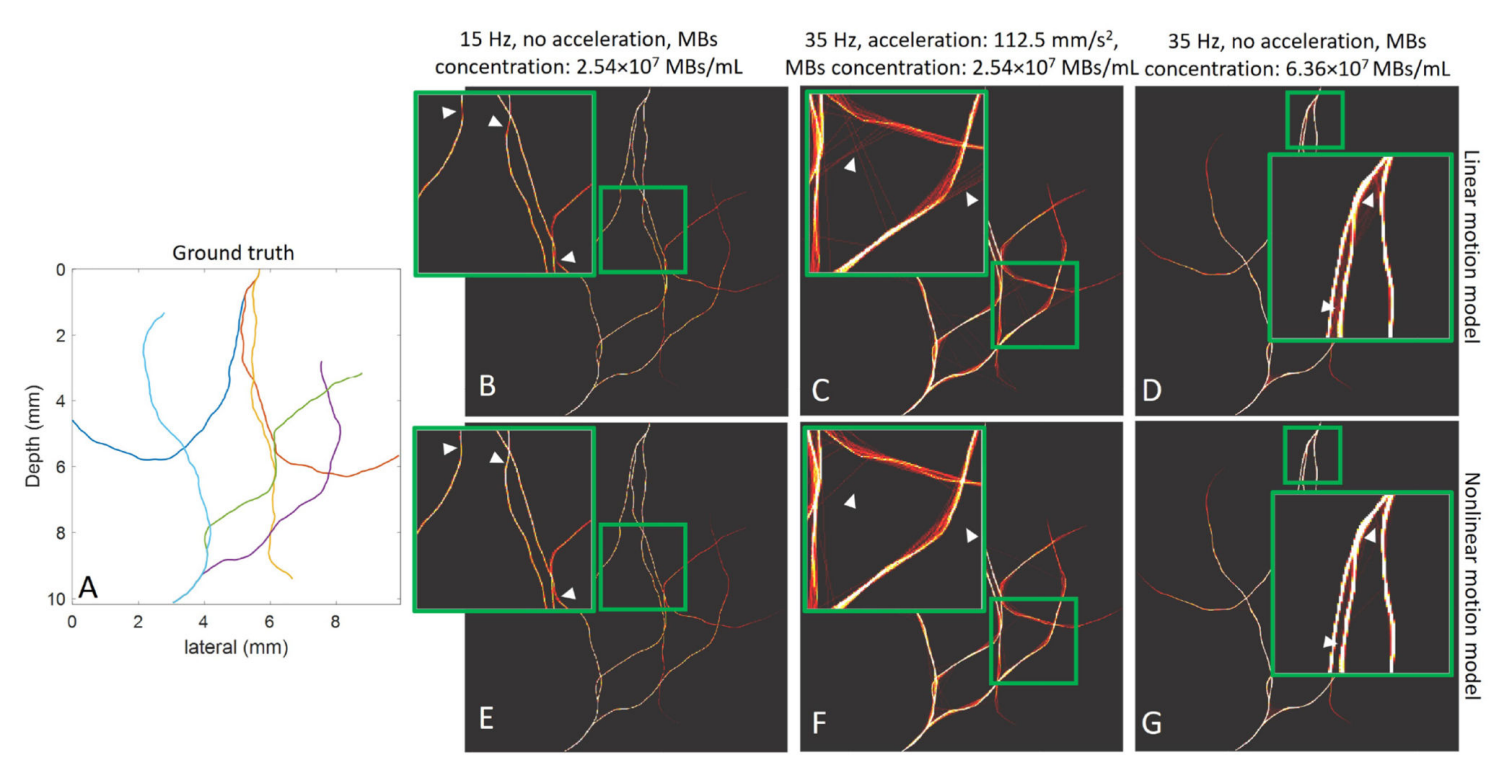
Visualisation of super resolution density maps at three simulation settings. Figure A: Ground truth vasculature. Figures B-C: Density maps from the linear motion model. Figures E-G: Density maps from the nonlinear motion model. The green boxes indicate the region of interest.

The visualisation of the tracking results is shown in Fig. 6. The ground truth of each vasculature is shown on the left. SR density maps reconstructed using the baseline linear motion model method and the proposed nonlinear motion model method are shown in B-D and E-G, respectively. Figures B and E correspond to the simulation with the lowest acquisition frame rate, acceleration, and MB concentration, while figures C and F correspond to the simulation with the highest acceleration and frame rate but lowest MB concentration. Figures D and G are results from the highest MB concentration, with the highest frame rate and lowest acceleration. In the first simulation, both methods showed high tracking precision when compared with the ground truth vasculature. However, the density map obtained with the baseline method displayed some dimmed segments in the zoomed-in region, indicating a higher pairing FNR compared to the proposed method. In the second simulation where the acceleration was set as the maximum, the density map from the proposed method showed a higher pairing TPR than the baseline method, as there were fewer incorrect links between the vessels. In the third simulation, the baseline method showed a lower pairing TPR compared to the proposed method, as observed from the blur between two closely situated vessels in figures D and G.

#### 2 Trajectory interpolation

**Fig. 7:**
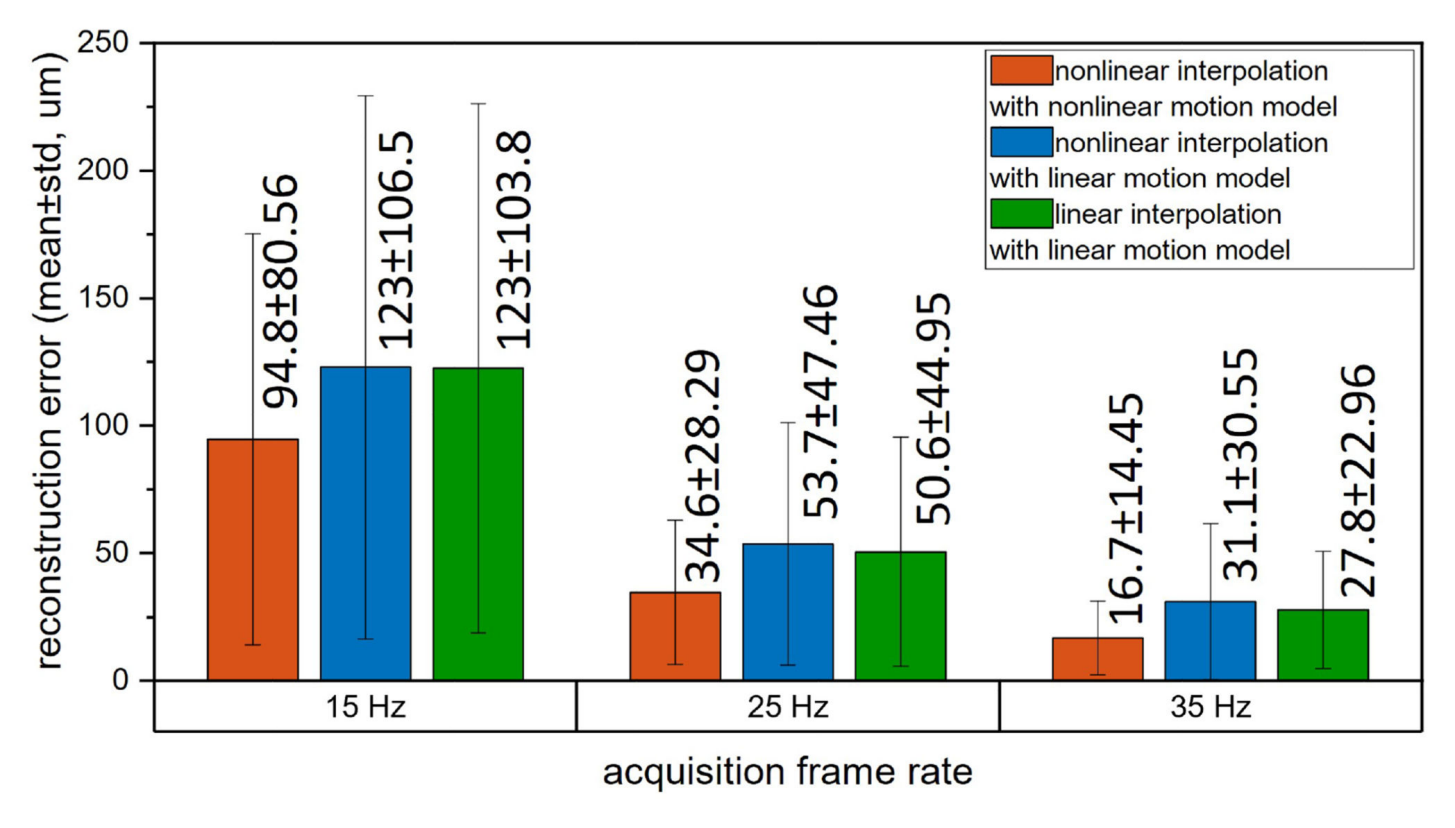
Comparison of interpolation errors among two nonlinear interpolation methods and a linear interpolation method at acquisition frame rates of 15, 25, and 35 Hz. Orange bar: nonlinear interpolation applied on acceleration-based nonlinear tracking results. Blue bar: nonlinear interpolation applied on linear tracking results. Green bar: linear interpolation applied on linear tracking results.

**Fig. 8:**
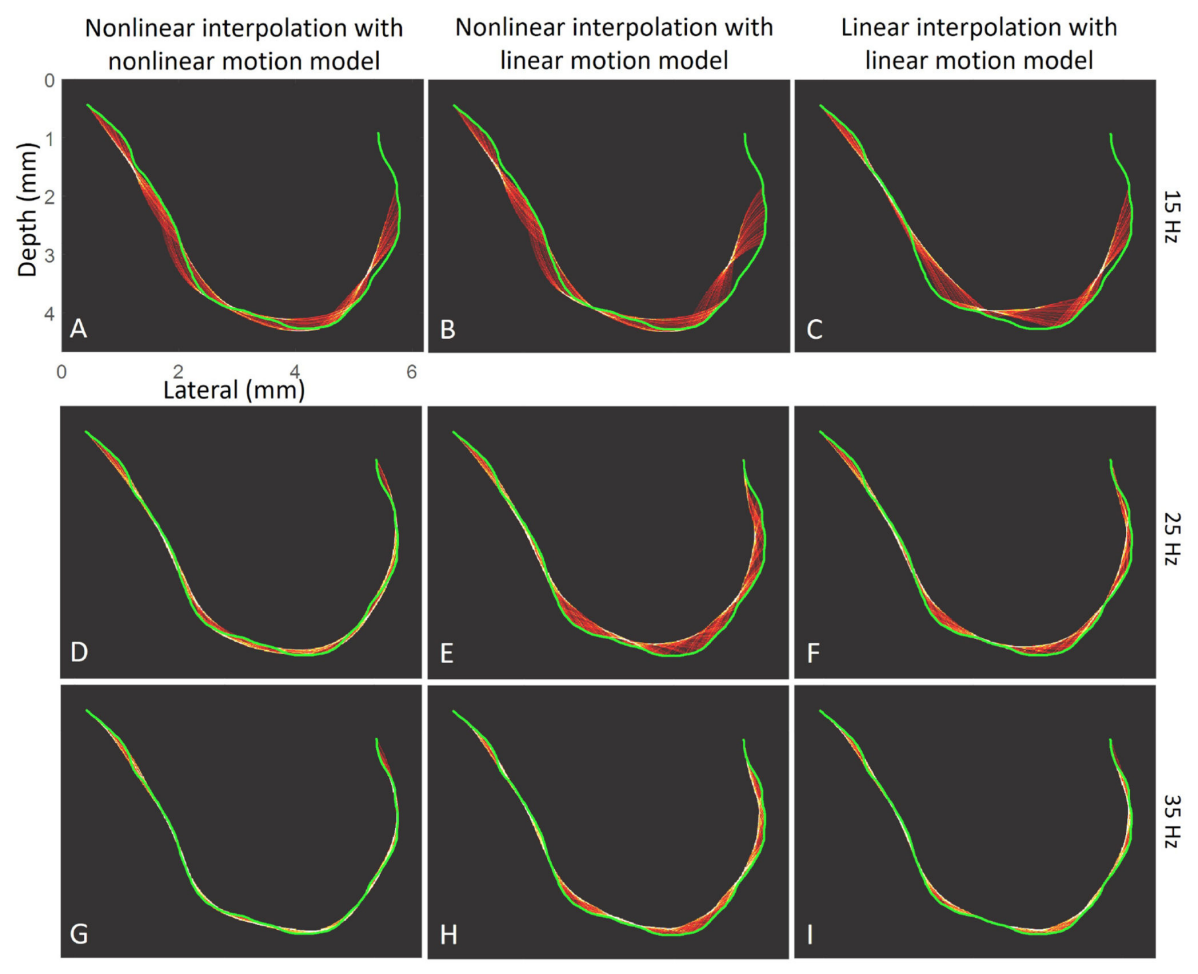
Examples of MB trajectory interpolation results using three interpolation methods. Column 1: Visualisation of MB trajectory reconstruction results using acceleration based-nonlinear interpolation on nonlinear tracking results. Column 2: nonlinear interpolation on linear tracking results. Column 3: linear interpolation results on linear tracking results. Row 1 to 3: simulations at acquisition frame rates of 15, 25, and 35 Hz.

The errors between the vessel structure and trajectories interpolated by the three aforementioned implementation methods are shown in Fig. 7. The pro-posed nonlinear interpolation, utilizing velocity estimates from a nonlinear motion model, exhibited the lowest average error. A visual comparison of the interpolation results is presented in Fig.8. Both methods yielded similar results in sections where the vessels are relatively straight. However, when the vessels were more tortuous, the nonlinear interpolation based on the nonlinear motion model outperformed the linear interpolation method in tracking the curved ground truth trajectory. In the interpolation simulation dataset, the average error of velocity estimation from the nonlinear model is lower than that from the linear model tracking (5.11 mm/s vs. 16.2 mm/s, 2.51 mm/s vs. 10.9 mm/s and 1.71 mm/s vs. 3.32 mm/s for 15 Hz, 25 Hz, 35 Hz, respectively).

### B In Vivo Experiments

**Fig. 9:**
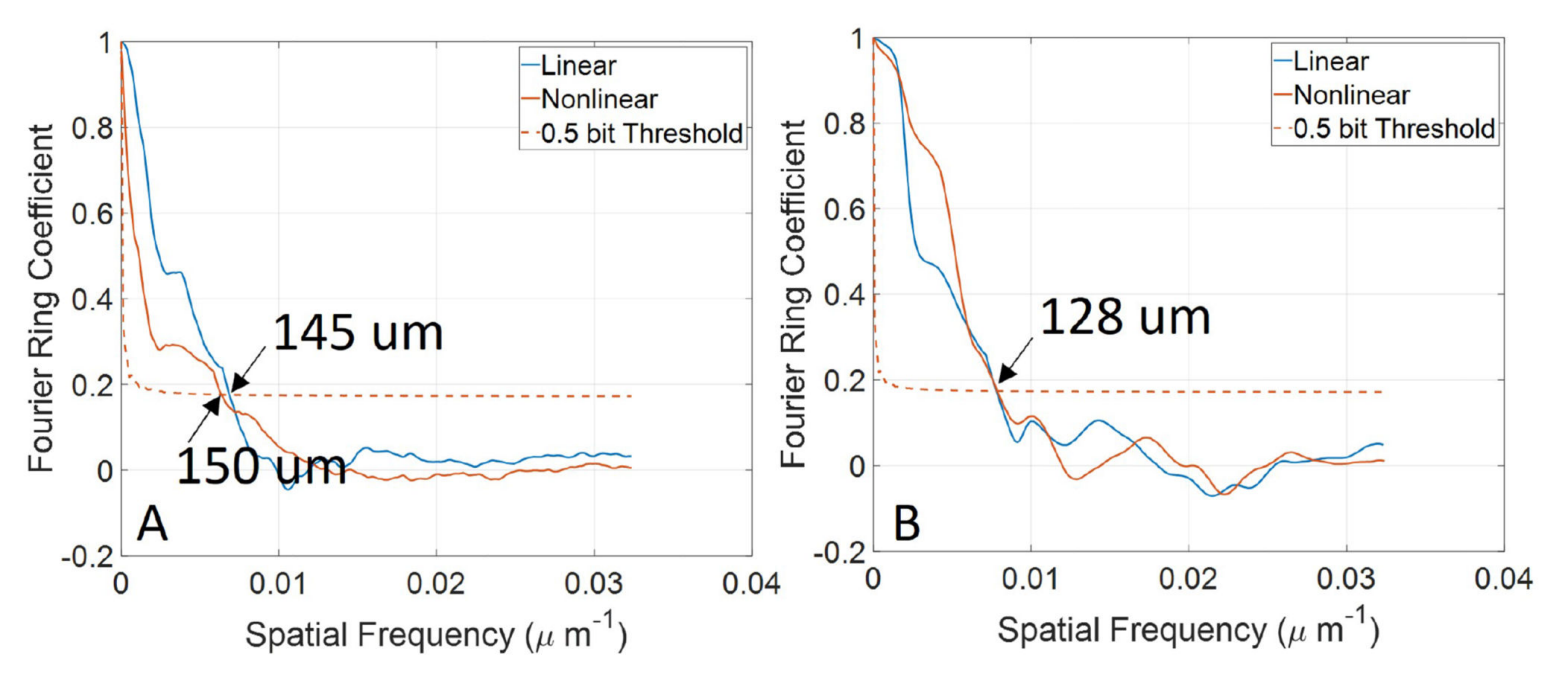
Estimation of imaging resolution by the Fourier ring coefficient method with a 1/2 bit threshold curve for *in vivo* dataset one (A) and dataset two (B).

**Fig. 10:**
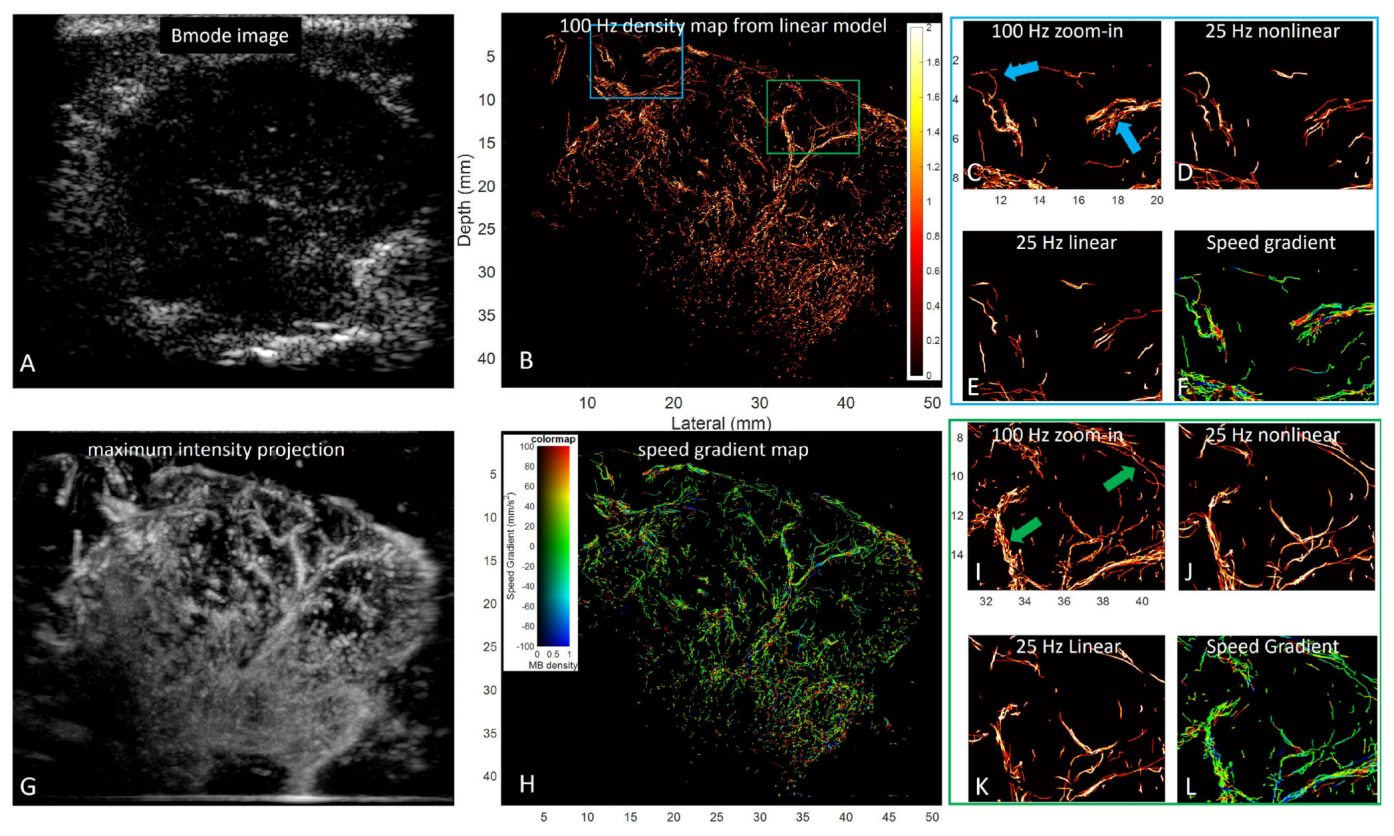
Comparison of super-resolution imaging results from the first *in vivo* dataset. (A): One frame of the B-mode images from the dataset. (B): MB density map obtained from the 100 Hz dataset with the linear model-based tracking method. (C): Magnified blue region of interest (ROI) in (B). (D): Density map obtained by the nonlinear model-based tracking method on the 25 Hz dataset in the same blue ROI as (C). (E): Density map obtained by the linear model-based tracking method on the 25 Hz dataset in the same blue ROI as (C). (F): Corresponding magnified speed gradient map in the blue ROI. (G): Maximum intensity projection of the original 100 Hz contrast-enhanced dataset. (H): A spatial speed gradient map generated from the nonlinear model-based tracking result. (I-L): Same layout of figures for the green ROI in (B). The colour bars denote the intensity of MB density and the speed gradient.

**Fig. 11:**
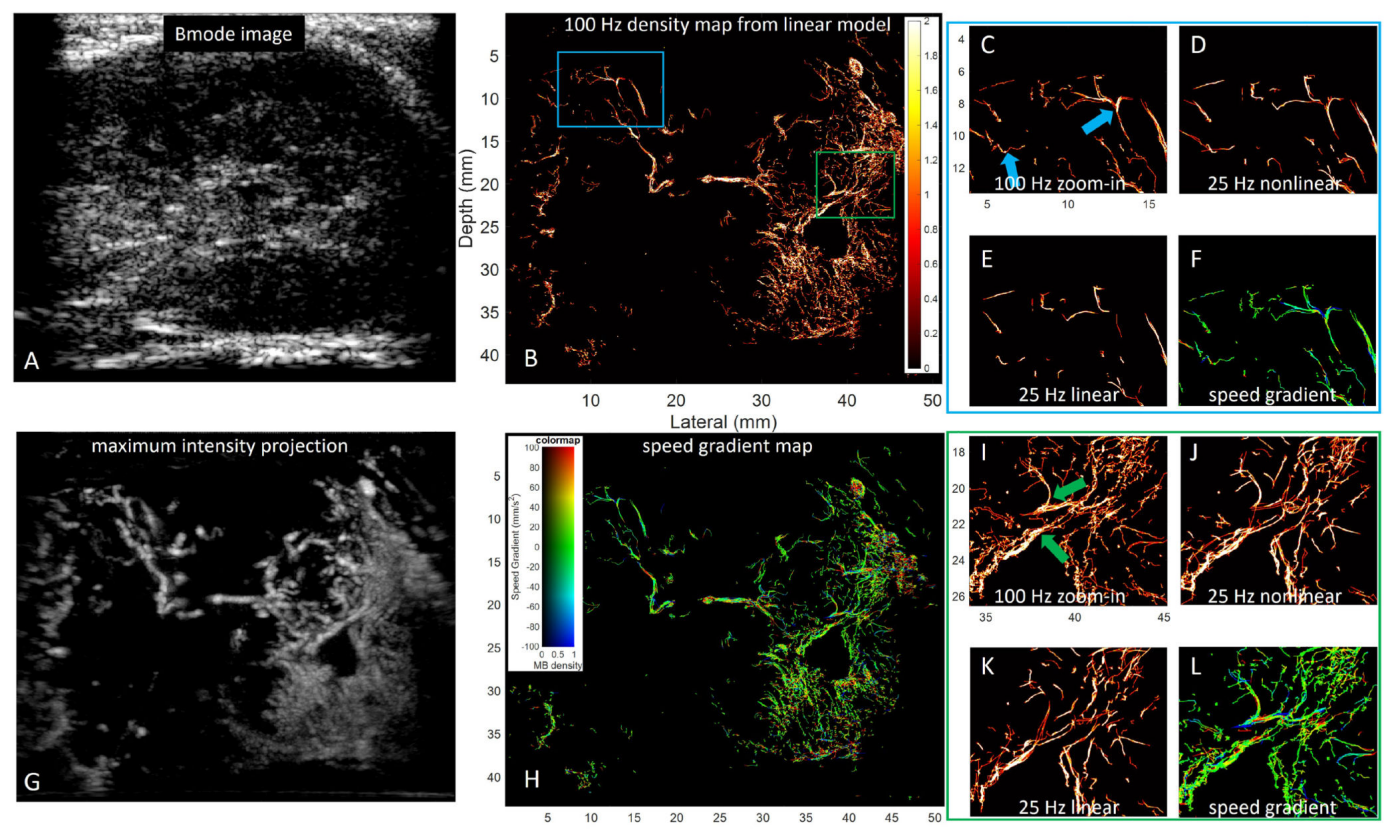
Super-resolution results from the second *in vivo* dataset. Captions are the same as in Fig. 10.

We reconstructed SR density maps of two 100 Hz frame rate datasets using both tracking algorithms respectively. The imaging resolution was estimated on the high frame rate dataset using the Fourier ring coefficient (FRC) method [8], [32], [33], as shown in Fig. 9. For dataset one, the resolution of the linear and nonlinear model-based tracking method was 145 *μ*m and 150 *μ*m, respectively. For dataset two, the resolution for both methods was 128 *μ*m. The estimated resolution exceeds the half wavelength of the transmitting ultrasound (154 *μ*m). The density maps reconstructed from full-frame-rate data by both algorithms show a high structural similarity index (calculated using the Matlab ‘ssim’ function): 0.98 for dataset one and 0.99 for dataset two. Therefore, we used the MB density map obtained from the 100 Hz dataset with the linear model-based tracking method as the reference for comparison with the reduced-frame-rate data, as shown in Fig. 10 and Fig. 11.

When we down-sampled the dataset 4 times, the proposed acceleration-based method outperformed the baseline method. From Fig. 10 and Fig. 11, more information is kept in the 25 Hz frame rate results of the proposed method than in the baseline method. The arrows in Fig. 10 and Fig. 11 highlight some differences in results from the proposed and baseline methods. For the proposed method, there was a higher consistency of vessels presenting in the 25 Hz and 100 Hz results indicating a better tracking performance. For dataset one, the proposed nonlinear method achieved an SSIM of 0.65 with the reference density map obtained from the 100 Hz dataset using the linear baseline method, while the baseline method achieved an SSIM of 0.63. For dataset two, the SSIM for the proposed method and the baseline method is 0.85 and 0.83, respectively. Compared to the MB density maps of the 100 Hz dataset, fewer MBs were tracked in the down-sampled 25 Hz dataset. Notably, the proposed method can better track MBs at positions with higher spatial speed gradients. Arrows in Fig. 10 highlight vessel branches that were reconstructed at 100 Hz but were missed by the baseline method at 25 Hz. Two curved vessels are presented with the proposed method in Fig. 10, while the baseline method failed to depict these vessels.

A spatial speed gradient map was generated for each of the datasets by averaging all the estimated speed gradients at the same position temporally, which is readily available after the proposed acceleration-based Kalman tracking. This information is in addition to MB density and flow velocity maps and may have diagnostic value.

## IV Discussion

### A Main Findings

In this study, we introduced an acceleration term into the Kalman-filtering-based MB tracking framework to improve MB tracking performance at low acquisition frame rates. Besides the MB tracking, incorporating acceleration also allows more accurate reconstruction of MB trajectories than linear interpolation. Results from simulation and *in vivo* experiments demonstrate improvement in MB tracking and vasculature reconstruction by the proposed methods. Additionally, a new type of super-resolution map, the spatial speed gradient map, is generated in this study to provide additional information.

The proposed algorithm was evaluated under different flow acceleration, MB density settings and acquisition frame rates, showing a consistently better tracking performance than the baseline. The constant acceleration assumption in the acceleration-based motion model becomes less reliable as the time interval between frames increases. Consequently, the improvement achieved with acceleration over methods without acceleration can be less significant when the frame rate is too low, as observed in the results at a frame rate of 15 Hz. In the performance comparison involving different flow accelerations, the nonlinear motion model inherently outperforms the linear model. When there is ”no acceleration” in moving speed, the acceleration still plays a role in changing the direction of MBs in curved vessels. The nonlinear model consistently demonstrates better performance with fewer missed pairs, as shown in Fig 6 (B and E), where there is no speed change in the simulation. When the acceleration is set to its maximum (112.5mm/s^2^), the TP, FP and TN still show significant improvement. The proposed method consistently outperforms the baseline method across various MB concentrations.

The proposed nonlinear interpolation method had a lower reconstruction error on average when compared with the linear interpolation. Although nonlinear interpolation can also be implemented with the baseline tracking method, the lower accuracy of velocity estimation results in more errors in acceleration estimation, which subsequently leads to greater misalignment in vessel reconstruction compared to the non-linear tracking method, as shown in Fig. 8. In addition, the proposed nonlinear interpolation with the nonlinear motion model might still result in significant errors if the frame rate is relatively low compared to the flow speed, as demonstrated in Fig. 7. This implies that a frame rate that is too low can reduce the image resolution when using MB trajectories to enhance image saturation.

From the *in vivo* studies, the performance between the proposed and baseline method has no significant difference when the frame rate is high, as it indicated by the high SSIM index. As it shown in the arrows in 10, the proposed method benefits MBs tracking in curved vessels. In Fig. 11, vessels with branches were pointed, where a higher magnitude of acceleration was also presented in the speed gradient map. From the corresponding speed gradient map, a higher magnitude of acceleration (more red or blue colour) can be observed at this branch. The spatial speed gradient visualisation proves the proposed method’s benefits of tracking the MBs with acceleration.

### B Difference from Previous Works

The nonlinear motion used for MB tracking tasks was mentioned in [24] to generate curved tracks. They modelled the MB movement with a constant speed and turning rate between frames. Compared with the linear motion model, their nonlinear model was a better approximation. An unscented Kalman filtering framework was used to incorporate their proposed nonlinear motion model [34]. In this paper, we proposed an acceleration motion model, approximating the MB movement as a curved motion with a changing speed. Compared with the linear motion model used in previous works, the acceleration term in the motion model avoided discontinuity in estimation of MB velocity in the tracking and is suitable for the scenario where pulsatile flow existed.

The Kalman filtering-based MB tracking framework was also used in others’ work. However, the initialisation of MB movement vectors has not been reported as far as we are aware. Inspired by the particle tracking velocimetry, we introduced a 3-frames-based MB motion state initialisation method for the first time in SRUS. A 3D graph-based algorithm was used to find the optimal initial pairing for new MBs.

The MBs’ trajectory reconstruction for super-resolution needs interpolation between linked MBs’ positions. To estimate the missing positions of MBs, linear interpolation was used in previous studies. Instead of using a fixed interpolation factor, an adaptive interpolation factor was also introduced by [19]. However, the hypothesis for linear interpolation that an MB kept moving with a constant velocity between frames may not hold in the case of low acquisition frame rates, tortuous vessels, and high flow speeds. In this study, we used nonlinear interpolation to reconstruct the trajectory and investigated the interpolation error between linear and nonlinear methods for the first time.

From our proposed MB tracking framework, we presented the spatial speed gradient maps for the microvasculature. The spatial gradient map may potentially indicate abnormal structural changes in the microvasculature, such as change of curvature or diameter, that results in a sudden change of MB movement.

### C Limitation and Future Work

In the MB motion state initialisation, we use a 3-frame-based method to estimate each newly appeared MB’s motion parameter. This proposed nonlinear-based method can be readily adapted to the 3D MB tracking application by adding an additional set of parameters to the Kalman state vector and covariance matrices for the elevational direction. It is worth exploring a 4-frame-based initialisation method, so the acceleration motion model can be implemented to estimate the MB’s state better. However, the higher computational cost of the 4D graph-based algorithm for 4-frame initialisation needs to be optimised in the future. The clinical application of the spatial speed gradient map from SRUS is also worth exploring.

## V Conclusion

In this paper, we introduced an acceleration-based motion model for MB tracking. A 3-frame-based motion state initialisation method was combined with an existing Kalman tracking framework. From the evaluation of both simulation and *in vivo* datasets, the proposed method is shown to improve MB tracking performance at low frame rates when there are tortuous vessels and accelerations in flow. The acceleration information can also be used for more accurate interpolation of MB trajectories between localised positions. Finally, a spatial speed gradient map is presented for the first time and could help explore the potential abnormal changes in the microvasculature.

## Highlights

- An acceleration-incorporated Kalman motion model is introduced into a microbubble tracking framework for microbubble tracking.
- The acceleration-based method outperformed the non-acceleration-based method in both microbubbles tracking performance and microvasculature reconstruction accuracy.
- The acceleration information can be used to improve super-resolution ultrasound imaging at low frame rates, when there are tortuous vessels and accelerations in flow.
